# Iconic faces are not real faces: enhanced emotion detection and altered neural processing as faces become more iconic

**DOI:** 10.1186/s41235-016-0021-8

**Published:** 2016-12-12

**Authors:** L. N. Kendall, Quentin Raffaelli, Alan Kingstone, Rebecca M. Todd

**Affiliations:** Department of Psychology, University of British Columbia, 2136 West Mall, Vancouver, V6T 1Z4 BC Canada

**Keywords:** Iconic faces, Face perception, Emotion, Expressions, P1, Event-related potentials

## Abstract

Iconic representations are ubiquitous; they fill children’s cartoons, add humor to newspapers, and bring emotional tone to online communication. Yet, the communicative function they serve remains unaddressed by cognitive psychology. Here, we examined the hypothesis that iconic representations communicate emotional information more efficiently than their realistic counterparts. In Experiment 1, we manipulated low-level features of emotional faces to create five sets of stimuli that ranged from photorealistic to fully iconic. Participants identified emotions on briefly presented faces. Results showed that, at short presentation times, accuracy for identifying emotion on more “cartoonized” images was enhanced. In addition, increasing contrast and decreasing featural complexity benefited accuracy. In Experiment 2, we examined an event-related potential component, the P1, which is sensitive to low-level visual stimulus features. Lower levels of contrast and complexity within schematic stimuli were also associated with lower P1 amplitudes. These findings support the hypothesis that iconic representations differ from realistic images in their ability to communicate specific information, including emotion, quickly and efficiently, and that this effect is driven by changes in low-level visual features in the stimuli.

**Electronic supplementary material:**

The online version of this article (doi:10.1186/s41235-016-0021-8) contains supplementary material, which is available to authorized users.

## Significance

Iconic images, which are symbols that resemble and simplify real-world stimuli, are ubiquitous in today’s society. They fuel profitable industries, such as the animation industry, and in the form of emoticons accompany every modern online and cellular communication tool available. Such a proliferation suggests the possibility that iconic images elicit facilitated cognitive processing, which endows them with enhanced communicative power. In contrast, in psychology, iconic faces are commonly used as easily controlled experimental stimuli with the unexamined assumption that they are functionally equivalent to real faces. Yet, outside of psychology, theories put forward by comic book artists themselves suggest this assumption is false. The research presented here draws on such ‘real-world’ understanding to suggest: (1) that factors conveyed by iconic representations of faces are not equivalent to those conveyed by real faces, and (2) iconic representations are more efficient at communicating emotional information. We found that increasing the level of abstraction of rapidly presented face stimuli by increasing simplicity and contrast improved the accuracy of emotion identification, consistent with an enhanced communicative role. Electrophysiological evidence further indicated that as images become more iconic due to changes in low-level features, emotional information is more rapidly transmitted to the viewer. Thus, the ubiquity of iconic imagery may be due to a unique capacity to facilitate communication of specific information. Such findings can contribute to our understanding of factors underlying the power and popularity of animation, scientific cartoons, and the emoticon; they may also encourage more informed use of schematized stimuli in psychology.

## Background

Iconic, or simplified, non-realistic images of faces are pervasive in popular culture and communicative media. Of the 100 highest grossing films of all time, one tenth were created using such iconic representations. The emoticon is included as a communicative tool used on every major online chat program distributed today. However, what advantages does an iconic representation have over a realistic one? Despite the ubiquity of iconic images, questions about their communicative function have been virtually ignored by cognitive science.

It is well established that real faces receive special treatment within our perceptual systems. For example, we are expertly tuned to recognize human faces and their expressions (Rhodes, Byatt, Michie, & Puce, 2004; Tsao, Freiwald, Tootell, & Livingstone, 2006), and we prefer looking at photographic faces over other stimuli (Chien, 2011). Yet, representations of faces also include cartoons, sketches, emoticons, etc., which can bear little resemblance to real faces, and these media seemingly have a niche in society that photorealistic stimuli do not fill.

Although neglected by cognitive psychologists, questions about the role of iconic imagery have been approached in other domains. For example, research has been conducted on narrative or syntactic structures underlying comic strips (Cohn, 2013). In the field of education, there has also been some research into the use of cartoons to improve scientific learning (e.g., Keogh, 1999; Naylor & Keogh, 2013). Outside of academe, in a popular graphic novel on comic analysis, McCloud (1994) has argued that the simplicity of iconic faces allows a media consumer to project themselves into a character. A potential clue to the differences between more realistic and iconic faces comes from the observation that simple representations of faces are often used in media created specifically for children. This may be because children rely more on low-level or simplified features characteristic of cartoons to process facial emotion (Gao, Maurer, & Nishimura, 2010). Additionally, individuals on the autistic spectrum are better at reading emotions on cartoon faces than on realistic faces (Rosset et al., 2008). Where the literature and the artist’s theory intersect is on the theme of simplicity – we propose that iconic faces use simplified and enhanced visual features to facilitate the communication of emotion.

Evidence that low-level visual features, such as contrast and complexity, influence identification of both facial identity and expression is consistent with this view. A large body of previous research suggests that face perception is heavily influenced by differences in stimulus type, especially in low-level visual features (Goffaux & Rossion, 2006; Crouzet & Thorpe, 2011; Sung et al., 2011; Yue, Cassidy, Devaney, Holt, & Tootell, 2011). For instance, low spatial frequencies that emphasize contrast provide an advantage in face identification (Halit, de Haan, Schyns, & Johnson, 2006), and high-contrast facial features elicit longer fixations than lower-contrast features (Neumann, Spezio, Piven, & Adolphs, 2006). Moreover, both contrast and spatial frequency profiles have been found to facilitate identification of fearful faces (Gray, Adams, Hedger, Newton, & Garner, 2013; Yang, Zald, & Blake, 2007).

If facilitated processing of iconic images is indeed predicted by low-level features, such as contrast and simplicity, underlying differences in cortical processing should be reflected in event-related potentials (ERPs). The P1 is an early perceptual ERP sensitive to low-level features in its latency and amplitude (Woodman, 2010; Kappenman & Luck, 2012). It is delayed by decreasing the luminance of a stimulus (Halliday, McDonald, & Mushin, 1973; Fimreite, Ciuffreda, & Yadav, 2015); it is delayed and lower in amplitude when a stimulus has lower contrast (MacKay & Jeffreys, 1973; Hosseinmenni, Talebnejad, Jafarzadehpur, Mirzajani, & Osroosh, 2015); and it is lower in amplitude for smaller relative to larger stimuli (Asselman, Chadwick, & Marsden, 1975). Early studies also found that stimuli with higher levels of pattern detail (i.e., finer checks on a checkerboard pattern) evoke larger P1 amplitudes than stimuli with larger low-level features, indicating a smaller amplitude P1 with reduced complexity (Lesèvre, & Rémond, 1972); Oken, Chiappa, & Gill, 1987; Zaher, 2012). Additionally, disorders that negatively impact low-level visual processing, such as multiple sclerosis, are associated with delayed P1 components (Halliday et al., 1973; Zaher, 2012). Together, these findings suggest that the P1 should be sensitive to clear and unambiguous features on a face. Specifically, as low-level features of an image become more cartoonized, i.e., simpler and higher in contrast, they should evoke a shorter latency and lower amplitude P1.

In contrast to the P1, the N170 is a face-sensitive ERP component modulated by emotional expression (Bentin, Allison, Puce, Perez, & McCarthy, 1996; Blau, Maurer, Tottenham, & McCandliss, 2007; Eimer, 2011; Hinojosa, Mercado, & Carretié, 2015). N170 amplitude and latency have been found to be sensitive to spatial frequency information (Halit et al., 2006) as well as contrast (Lu, Wang, Wang, Wang, & Qin, 2014) and image complexity (Churches, Nicholls, Thiessen, Kohler, & Keage, 2014). Despite these established findings, an open question concerns whether amplitude or latency patterns observed in the P1, due to increased simplification of cartoon images, are carried on to the N170.

Convergent findings suggest that this may be the case. Although the P1 is neither a face-specific nor emotion-sensitive component, there is some evidence that it is the first component that is sensitive to differences in face stimuli in childhood. In contrast, the N170 develops face sensitivity later in development (Taylor, Batty, & Itier, 2004). These findings support the conclusion that low-level features in faces play a greater role in face discrimination earlier in development, and face-specific processing is built on this detection of basic visual features. Such findings are also consistent with the notion that the P1 plays a role in face detection without requiring sensitivity to faces as a specific category. Moreover, unlike the N170 (Desjardins & Segalowitz, 2013; Rossion & Caharel, 2011), the P1 is modulated by the presence or absence of face features even in scrambled faces. Thus, convergent evidence indicates that the P1 reflects cortical activity implicated in early feature detection, which contributes to face sensitive processing indexed by the N170. Although the holistic processing that is distinctive of face processing may not occur prior to the N170 (Rossion, 2014), this earlier component may influence later face processing by allowing for faster processing if features have been more quickly recognized.

In sum, previous research suggests that face perception is heavily influenced by differences in stimulus type, especially in low-level features, and that such manipulations modulate rapid cortical activity that precedes holistic face perception. Thus, enhanced communicative capacity through intensification and simplification of low-level features in iconic images, such as emoticons and cartoons, may underlie their ubiquity, despite their seeming dissimilarity from real-world stimuli. The present study tested the hypothesis that, as faces become more iconic, emotional information becomes easier to access. We thus created face stimuli increasing in schematization through simplification (reduced complexity) and enhancement (higher contrast). In Experiment 1, we examined whether schematization affected the detection of emotional expressions across a range of presentation times. We predicted that accuracy would increase with schematization, and that this would become more pronounced as presentation time decreased. In Experiment 2, we used ERPs to examine how neural responses to manipulations of complexity reflected schematization advantages in emotion detection. We predicted differences in amplitude and latency with simplification that would correspond with our behavioral results.

### Experiment 1

#### Participants

A total of 50 undergraduates (36 female, mean age = 20; 14 male, mean age = 20.8) participated for course credit. A sample size of 25 participants per level for each between-subject independent variable was determined before data collection as sufficient to find meaningful effects based on previous studies done by our research group. The present number was determined as the experiment included a comparison of mixed versus blocked trials as the only between-subject factor. Data collection ceased when 50 participants had been tested.

## Methods

**Fig. 1:**
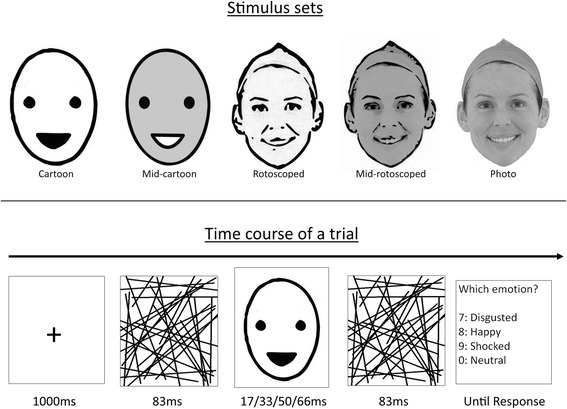
An example of the five stimulus sets used and a time course of a single trial for Experiment 1. The “Cartoon” and “Mid-cartoon” stimulus sets have less complex features than the “Rotoscoped” and “Mid-rotoscoped” sets, and the “Cartoon” and “Rotoscoped” stimulus sets are higher in contrast than the “Mid-cartoon” and “Mid-rotoscoped” sets. Photos may have other low-level featural differences in addition to contrast and featural complexity, but are used here as a baseline non-schematic condition

Figure 1 illustrates the stimuli, which consisted of five categories of faces employing increasing degrees of schematization: cartoon (non-realistic iconic faces where the only features present are used for communication, such as the eyes and mouth), mid-cartoon (the same as cartoon, but with a “skin tone”, so that the face has a shade of grey darker than the white background), rotoscoped (photographs that have been schematized by using a technique for drawing over the photograph, creating a heavy outline to emphasize high contrast features like the eyes, mouth, and nose, while removing others), mid-rotoscoped (the same as rotoscoped but leaving the average skin-tone of the photo intact so that the face is darker than the white background), and unmanipulated realistic photos, which acted as a non-schematized control or baseline stimulus set. Each stimulus group was comprised of an equal number of images.

The cartoon and mid-cartoon stimuli were constructed using basic photo manipulation software, GIMP (Kimball & Mattis, 1996). The positions of features were varied slightly from image to image in the mid-cartoon set so that there were several versions of each face (e.g., the eyes could be shifted to be slightly wider, so that not every cartoon face is perfectly identical), and then the ‘skin-tone’ was removed (i.e., turned white) from each of these to create the cartoon stimulus set. To create the rotoscoped and mid-rotoscoped stimulus sets, the photo stimulus set was processed using rotoscoping software by Synthetik (Dalton, 1999). In this way, in addition to the realistic photos, which acted as a non-schematic control, we created four stimulus sets which non-linearly spanned a range from realistic to schematic (i.e., cartoon) faces. As a manipulation check, we asked another group of 60 participants to rank order the stimulus sets from least realistic to most realistic; 70 % ordered them in the order illustrated above (the second most common configuration being a simple switch of the mid-cartoon and rotoscoped sets, which comprised 20 % of responses).

With the photo stimuli excluded, these stimuli can also be grouped by two factors, contrast and featural complexity. The mid-rotoscope stimulus is identical to the rotoscoped stimulus set, but with lower contrast, and the mid-cartoon is identical to the cartoon stimulus set but with lower contrast. Likewise, the cartoon and mid-cartoon stimulus sets can be seen as less featurally complex versions of the rotoscoped and mid-rotoscoped stimulus sets, respectively. Further discussion of this is provided below in the secondary analysis of Experiment 1.

**Fig. 2:**
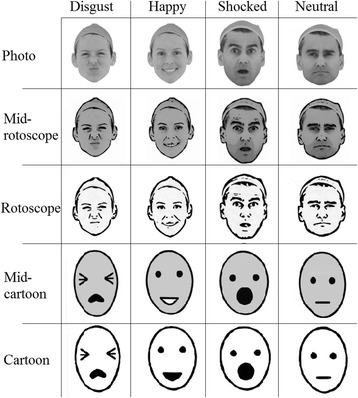
An example of each type of stimulus set for each type of emotional expression used

Each stimulus set included four categories of facial expression: disgusted, happy, surprised, or neutral. These expressions were selected as commonly recognized basic emotions (e.g., Ekman, Sorenson, & Friesen, 1969) that were physically distinct from each other and of mixed valence. Because surprise can be either negative or positive, we used the term “shocked” to emphasize negative valence and reduce participant confusion. There were eight variants of each expression in each set, represented by different individuals’ faces in the more realistic sets, and by varied feature positions in the cartoon and mid-cartoon sets. Thus, there were 160 images in total (8 variants × 4 expressions × 5 stimulus sets). For the purpose of rotoscoping, we used faces from a database of emotional expressions created to be used for animation. See Fig. 2 for an example of each emotional expression for each stimulus set.

All images were presented using PsychoPy software (Peirce, 2007). Each of the 160 images was shown four times, corresponding to four possible presentation times. The four presentation times were 16.7, 33.3, 50, and 66.7 ms, which correspond to 1, 2, 3, and 4 frames on a monitor with a 60 Hz refresh rate, and were chosen randomly on a trial by trial basis. Therefore, in total, there were 640 trials (160 images × 4 presentation times).

A further manipulation concerned presentation of stimulus type in a blocked (i.e., all cartoon images together, all photos together) or event-related design (any stimulus type could be chosen for each trial, randomly). However, there was no statistical difference between this between-subjects factor (*P* = 0.71), and was therefore not further considered.

### Procedure

All participants performed the task on a laptop in a dimly lit testing room. Images were displayed on a neutral grey background (125, 125, 125 in the RGB color system).

Figure 1 illustrates the sequence of events in a typical trial. Before each trial, a fixation point (+) was positioned on the middle of the screen, and participants were told to keep their eyes on its location. A random line mask would appear for 83 ms (i.e., 5 frames out of 60), then one of the face images at one of the four presentation times, and then another mask, followed by a response screen. Stimuli were forward and backward masked to ensure that they were only visually discernable for precise presentation times. Participants were asked to identify the expressions of the images presented to them, as quickly and as accurately as possible, using numbered keys: 7, 8, 9, and 0, representing disgusted, happy, shocked, and neutral, respectively. The next trial was presented following the previous response (i.e., as there was 1000 ms of fixation preceding each stimulus presentation, the intertrial interval was always 1000 ms).

Subjects were given a practice session with feedback at the beginning of the experiment to familiarize them with the program before the actual recorded trials. Subjects were also given the option of resting breaks every 128 trials. Accuracy was recorded for each trial.

## Results

**Fig. 3:**
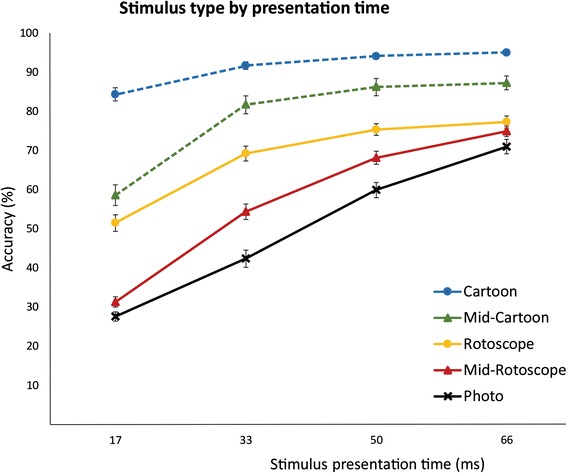
Accuracy rate for five stimulus categories at each presentation time for Experiment 1. Error bars represent standard error of the mean. Dotted lines denote low featural complexity stimuli, with solid lines as high featural complexity stimuli. Circle markers denote high contrast stimuli, with triangles as low contrast stimuli. Photo, the baseline stimulus set, in marked in black. Chance is at 25 %

Figure 3 shows all stimulus sets at each presentation time. A 5 × 4 × 4 (level of schematization × expression × presentation time) repeated measures ANOVA was conducted to analyze accuracy data (there were insufficient correct trials in each condition for response time to be a meaningful measure, and so no analyses on response time data are included here). Although expression was included as a factor in analysis, there were no meaningful interactions with presentation time or stimulus type (i.e., all significant results trended towards disgust simply showing a slightly more exaggerated pattern of results, and neutral expressions showing an attenuated pattern of results, while always showing the same order of detectability for each stimulus type). Thus, expression results will not be reported here; however, all values for all conditions can be found in Table 1.

**Table 1:** Full accuracies for Experiment 1

			Presentation time (ms)			
			17	33	50	66
Photo	Disgust	Mean	0.173	0.267	0.483	0.600
		Std. Error	0.024	0.032	0.042	0.038
	Happy	Mean	0.171	0.481	0.685	0.827
		Std. Error	0.025	0.041	0.034	0.026
	Shocked	Mean	0.223	0.507	0.719	0.810
		Std. Error	0.038	0.040	0.030	0.032
	Neutral	Mean	0.529	0.424	0.485	0.569
		Std. Error	0.044	0.036	0.036	0.030
Mid-rotoscoped	Disgust	Mean	0.234	0.437	0.559	0.612
		Std. Error	0.028	0.036	0.036	0.035
	Happy	Mean	0.234	0.558	0.786	0.882
		Std. Error	0.032	0.032	0.023	0.019
	Shocked	Mean	0.266	0.658	0.789	0.815
		Std. Error	0.036	0.039	0.024	0.026
	Neutral	Mean	0.522	0.508	0.582	0.677
		Std. Error	0.041	0.034	0.033	0.029
Rotoscope	Disgust	Mean	0.506	0.588	0.678	0.655
		Std. Error	0.041	0.041	0.032	0.029
	Happy	Mean	0.547	0.730	0.861	0.886
		Std. Error	0.034	0.026	0.020	0.019
	Shocked	Mean	0.491	0.755	0.729	0.779
		Std. Error	0.032	0.035	0.030	0.030
	Neutral	Mean	0.513	0.691	0.740	0.776
		Std. Error	0.037	0.031	0.025	0.029
Mid-cartoon	Disgust	Mean	0.577	0.777	0.807	0.803
		Std. Error	0.042	0.035	0.036	0.033
	Happy	Mean	0.543	0.786	0.854	0.863
		Std. Error	0.040	0.034	0.033	0.032
	Shocked	Mean	0.620	0.846	0.879	0.881
		Std. Error	0.036	0.027	0.023	0.020
	Neutral	Mean	0.581	0.871	0.912	0.943
		Std. Error	0.043	0.021	0.021	0.013
Cartoon	Disgust	Mean	0.870	0.922	0.939	0.946
		Std. Error	0.022	0.016	0.017	0.014
	Happy	Mean	0.903	0.948	0.961	0.965
		Std. Error	0.019	0.011	0.011	0.009
	Shocked	Mean	0.861	0.920	0.931	0.931
		Std. Error	0.027	0.021	0.015	0.017
	Neutral	Mean	0.739	0.886	0.939	0.960
		Std. Error	0.035	0.022	0.016	0.011

All contrasts were Bonferroni corrected for multiple comparisons. Data from participants who scored 2.5 standard deviations above or below the mean accuracy were discarded. Data from two participants were excluded based on this criterion (both had overall accuracies lower than 40 %, with some conditions having 0 %). Data from an additional participant were excluded due to a program malfunction, resulting in 47 participants being included in the final analysis. When necessary, F values were subjected to the Huynh–Feldt correction for the violation of the assumption of sphericity.

First, there was a main effect of presentation time [F(3,138) = 753.24, *P* < 0.001, η^2^_p_ = 0.94], with accuracy increasing as presentation time increased. There was a significant main effect of level of schematization [F(4,184) = 241.68, *P* < 0.001, η^2^_p_ = 0.84], indicating overall differences between levels of schematization. Follow-up comparisons revealed that each stimulus type was significantly different from all the others (all *P* < 0.001), with highest accuracy for cartoons, followed by mid-cartoon faces, rotoscoped faces, mid-rotoscoped faces, and photos. This pattern of results supported our prediction that images become easier to process as they become schematized.

Crucially, there was an interaction between level of schematization and presentation time [F(12, 552) = 29.49, *P* < 0.001, η^2^_p_ = 0.39]. To further probe this interaction, each stimulus set was compared in a separate ANOVA at each presentation time, with significance levels Bonferroni adjusted for multiple comparisons. For brevity, only significant differences are reported.

At 17 ms, accuracy for cartoon stimuli was higher than for all other stimulus categories. Accuracy for mid-cartoon and rotoscoped images was lower than for cartoon images, but higher than for photo and mid-rotoscoped images (*P* < 0.001). At presentation times of 33 ms and 50 ms, all stimulus sets differed from one another (*P* < 0.01), with highest accuracy for cartoon images and lowest for photographic images. Finally, at the presentation time of 66 ms, of the three lowest accuracy stimulus sets (photo, mid-rotoscoped, and rotoscoped images), only accuracy for rotoscoped images and photos differed from each other.

Thus, the interaction revealed both a sharper increase in accuracy for the mid-rotoscoped and mid-cartoon stimulus sets between presentation times 17 ms and 33 ms, and a general leveling out of accuracies at 66 ms. However, it is important to note that the order of conditions from highest accuracy to lowest was the same at all presentation times: cartoon, mid-cartoon, rotoscoped, mid-rotoscoped, and photo.

The pattern of accuracy differences between stimulus types, particularly at the shortest presentation times, suggested the possibility that two low-level features, contrast and complexity, were contributing to accuracy of expression identification in ways which could easily be dissociated.

In a follow-up analysis, we collapsed across emotion and presentation time in all stimulus types except photographs. Within the remaining stimulus categories, the mid-rotoscope stimulus is a low-contrast version of the rotoscope stimulus, and the mid-cartoon a low contrast version of the cartoon stimulus. That is, the contrast ratio (calculated as the relative RGB luminance of the lightest color + 0.05/the relative luminance of the darkest color + 0.05) between the face outline and features and the face ‘skin-tone’ in the rotoscope and cartoon stimuli is always 21:1, or the maximum contrast on a computer monitor (i.e., pure black on pure white); the average contrast ratios for the mid-rotoscope and mid-cartoon stimuli are 7.5:1 and 12.8:1, respectively.

**Fig. 4:**
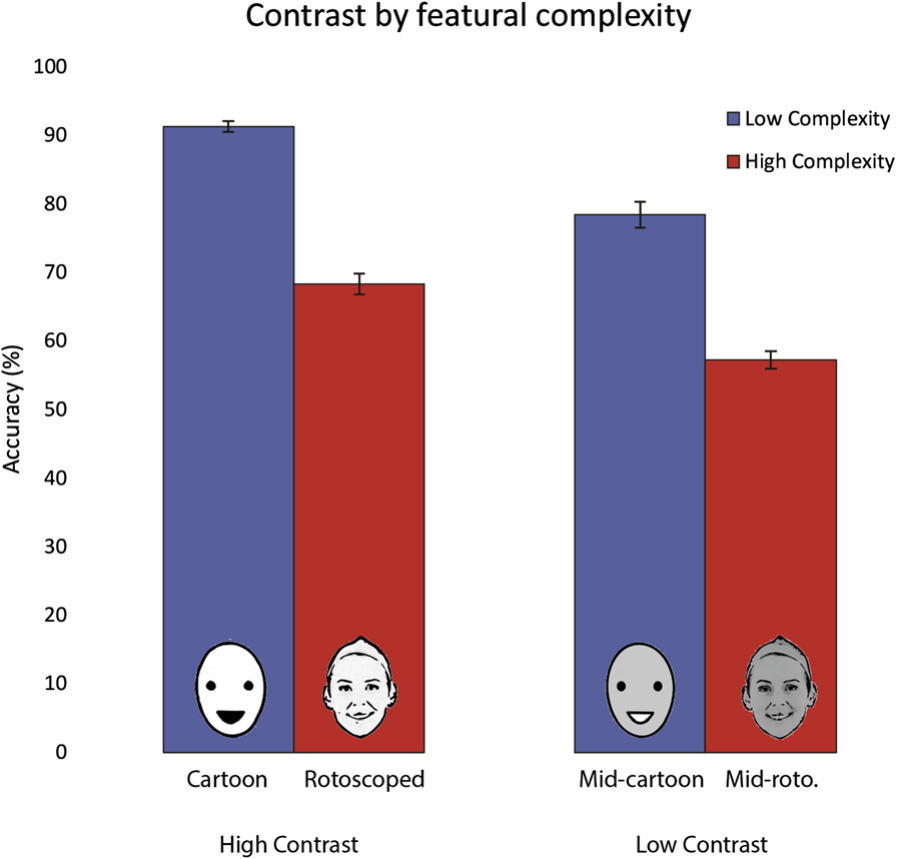
Accuracy for all but the photorealistic photo sets in Experiment 1, arranged to illustrate the separate effects of contrast and featural complexity. Error bars represent standard error of the mean

Likewise, the two cartoon stimulus types (cartoon and mid-cartoon stimuli) can be seen as lower featurally complex versions of the non-cartoon stimulus types (Fig. 4). To confirm this, we tested a separate sample of 20 participants on how complex or simple each of our image sets were using a Likert scale from 1 (least featurally complex) to 7 (most featurally complex). The results of this manipulation check confirm that the mid-cartoon (M = 1.91, SD = 1.38) and cartoon (M = 1.73, SD = 1.24) sets were in fact perceived as less complex than the mid-rotoscoped (M = 4.50, SD = 1.54) and rotoscoped sets (M = 3.77, SD = 1.59).

We thus grouped the mid-rotoscoped and mid-cartoon stimulus sets as “low contrast”, with the rotoscoped and cartoon as “high contrast”. Similarly, cartoon and mid-cartoon images were grouped as “low featural complexity” and rotoscoped and mid-rotoscoped images as “high featural complexity”. In this way, we could compare the main effect of contrast and the main effect of featural complexity independently across our stimulus sets, using a 2 × 2 repeated measures ANOVA.

This analysis found a main effect of contrast [F(1,46) = 116.69, *P* < 0.001, η^2^_p_ = 0.72], as well as a main effect of featural complexity [F(1,46) = 408.77, *P* < 0.001, η^2^_p_ = 0.90], but no interaction between them (*P* > 0.250). This suggests that both the contrast and the complexity of an image affect accuracy (with higher contrast and lower complexity being represented as higher accuracies), but they do so without interacting with one another.

To examine the potential effects of presentation time on our results, we next performed an ANOVA that included presentation time as an additional factor (three within-subject factors: contrast, featural complexity, and presentation time).

Here, there were interactions between presentation time and contrast [F(3,144) = 61.93, *P* < 0.001, η^2^_p_ = 0.56], and presentation time and complexity [F(2.26,106.26) = 23.04, *P* < 0.001, η^2^_p_ = 0.32], as well as an interaction between all three factors [F(2.11,101.49) = 5.78, *P* < 0.001, η^2^_p_ = 0.11].

Follow-up comparisons for the presentation time by contrast interaction revealed that the interaction was driven by an attenuation of differences within a category type at longer stimulus presentation times. Critically, all comparisons of high contrast and low contrast images were significant at every presentation time (*P* < 0.005). The interaction was instead driven by the comparison of the high contrast images at 50 ms and 66 ms presentation times for *P* = 0.267. That is, there was no difference in accuracy between these two presentation times, but only for high-contrast images.

For the presentation time by complexity interaction, a similar pattern was found, where all comparisons of complexity at each presentation time were significant (*P* < 0.001), so that high complexity images were always different from low complexity images at every presentation time. Instead, the interaction was driven by the comparisons of the longest two time windows (50 and 66 ms) for the low complexity images. In sum, these interactions show that features which promote discrimination of images, namely high contrast or low complexity, show less of a benefit at longer presentation times, and so may have hit a “ceiling” of their usefulness between 50 and 66 ms presentation times. However, the influence of contrast and featural complexity were always significant at each presentation time.

This levelling-off effect within a stimulus set also characterized the three-way interaction. Comparisons between levels of contrast or levels of complexity were always significant at every presentation time (*P* < 0.001). As with the previous interactions, this interaction was driven by a comparison of presentation times within stimulus sets. If the image was either high contrast or low complexity or both, the comparison of 50–66 ms time windows was non-significant. In the stimulus set with high contrast and low complexity, the comparison between the 33 ms and 50 ms presentation times was also non-significant. Essentially, this reflected a combination of what was found in the separate two-way interactions – for features that promote discrimination of images (high-contrast or low featural complexity), there is less of a benefit at longer presentation times. However, just as in the smaller interactions, the influence of contrast and featural complexity were always significant at each presentation time.

In sum, these results confirmed our prediction that images become easier to process as they become schematized – specifically as they become less complex and higher in contrast. Given that both complexity and contrast influenced emotion detection in a dissociable manner, and that contrast has been previously shown to decrease ERP latencies (Lu et al., 2014), in the next study, we focused on complexity using ERPs to further examine the influence of feature simplification on facial emotion processing.

### Experiment 2

#### Participants

A total of 29 participants were recruited from the UBC psychology human subject pool (22 female, mean age =19.9; 7 male, mean age = 20.1). Here, again based on past studies performed by our research group, we considered a sample size of 25 as sufficient for finding meaningful ERP results. Four extra participants were included because they had already signed up to participate in redundant timeslots on the final week of testing (i.e., we concluded data analysis at the end of the week of the 25th participant). Two participants were excluded due to excessive electroencephalography (EEG) artifacts or poor performance. As with the previous experiment, participants were given course credit in exchange for participation.

### Methods

All stimuli were identical to those in experiment one, except that the mid-cartoon and mid-rotoscoped stimulus sets were discarded for two reasons, namely to maximize the number of trials recorded in ERP version of the task and to focus on the influence of contrast and complexity in facial emotion detection.

This left three stimulus sets – photo, rotoscoped, and cartoon faces. Whereas the comparison of cartoon with rotoscoped and photo stimulus sets would reveal the impact of featural complexity on face processing, the comparison of rotoscoped and photo images would demonstrate effects of contrast.

All images were also presented in blocks, so that between breaks only one stimulus set was shown at a time. In addition, we eliminated the use of masks to allow for a clean ERP response, and all stimuli were presented for 500 ms to ensure that a complete waveform would be detected. To allow sufficient trials for ERP averaging, we also presented more trials for each stimulus type than in Experiment 1. Here, 360 trials of each stimulus type were presented in randomized blocks of 120 trials each, over the course of an hour for a total of 1080 trials.

The task participants were given was identical to the behavioral task. Participants responded to which emotion (happy, shocked, disgust, neutral) was expressed by each face after it was presented. However, to prevent response noise in the EEG data, participants were told not to respond until they reached the response screen, which occurred immediately after the 500 ms presentation time of the stimulus, although they were told to answer as quickly and accurately as possible once the response screen was shown. Due to the 500 ms presentation time compared to the 17–66 ms presentation times of the behavioral task, this task was designed to be much easier.

### EEG data acquisition

Scalp recorded EEG data was recorded using a 64 channel Biosemi Actiview system. All EEG data was processed using the Matlab toolbox, ERPLAB (Lopez-Calderon & Luck, 2014). Continuous EEG data was recorded at a sampling rate of 512 Hz, and was band-pass filtered using an IIR Butterworth filter with half-amplitude cut-offs at 30 and 0.1 Hz during offline preprocessing. All data were referenced to two additional electrodes, which were placed on the left and right mastoid processes. In addition, two electrodes were placed on the outer canthi of both eyes, and one electrode was placed below the right eye. These three electrodes were used to record eye movement and blink information so that trials containing eye movements could be discarded.

Epochs for sectioning continuous data into ERP bins were 500 ms in length, time-locked to stimulus onset, and were referenced to 200 ms pre-stimulus onset. Data was processed automatically for artifacts using a moving window peak-to-peak method with the epoch length as the test period and window of 100 ms, resulting in less than 10 % of total trials being rejected. Finally, data from correct responses for each type of trial (e.g., disgust cartoon, disgust photo, etc.) were averaged together for each participant.

Peak P1 activation was extracted from each averaged epoch by measuring local peak amplitude at electrode Oz and local peak latency within a window of 80-160 ms after stimulus onset. If no peak was reliably found using this method, that participant was excluded. This resulted in two participants being excluded from P1 analyses, leaving 25 participants.

For a P1-N170 peak-to-peak analysis described below, the P1 and N170 were extracted using local peak amplitude and local peak latency at electrodes P9 and P10 within a window of 150–220 ms (see Additional file 1 for N170 results). For this analysis, two participants who did not have P1s or N170s that could be reliably extracted were excluded, leaving a total of 25 participants.

### EEG analysis

#### P1

The P1 is observed at posterior sites, and is typically maximal at electrodes contralateral to where an attended stimulus was presented in the visual field (e.g., Luck, Heinze, Mangun, & Hillyard, 1990). As our stimuli were presented directly in the center of the visual field, we performed our initial P1 analyses on electrode Oz, which was also the electrode that showed the highest P1 amplitude in a grand average across all participants and conditions.

#### Peak-to-peak analysis

A further question concerned whether differences between conditions reflected processes specific to face processing (i.e., represented by differences in the N170 independently from the P1) or simply low-level featural changes in our stimulus sets carried over into the N170. For this secondary analysis, we next performed a peak-to-peak comparison to determine if the earlier P1 component was contributing to the N170 at the same electrode sites. Here, P1 and N170 peaks were extracted at sites where the N170 was maximal. The N170 ERP component is typically strongest at six occipito-temporal electrodes (P7, P9, P07, and P8, P10, P08) (Sagiv & Bentin, 2001). However, only the most ventral two of these electrodes allowed reliable extraction of N170 and P1 peaks, namely electrodes P9 and P10. Although all six electrodes listed above are common targets for N170 peak extraction, it is also common to find the clearest or strongest N170 at P9/P10 (e.g., Itier, Van Roon, & Alain, 2011; Fisher, Towler, & Eimer, 2015). Thus, our peak-to-peak analyses were performed on data extracted only from these two electrode sites.

ERP epochs were averaged for each stimulus type (cartoon, rotoscoped, photo), for each expression (disgusted, happy, shocked, and neutral), and for the three electrode sites (Oz, P9, and P10).

### Results

#### Behavioral data

The 3 × 4 (stimulus type by emotional expression) repeated measures ANOVAs were performed on both accuracy and reaction time data. For accuracy, there was a main effect of stimulus type [F(1.4,39.14) = 40.63, *P* < 0.001, η^2^_p_ = 0.59]. Follow-up comparisons revealed a greater accuracy for cartoon faces (98.2 %) than for rotoscoped (93.4 %) (*P* < 0.001) or photo (94.0 %) (*P* < 0.001) images. The difference between rotoscoped and photo images, as a measure of contrast, was not significant (*P* = 0.28), suggesting lower contrast on its own did not facilitate performance.

For reaction time, there was a main effect of stimulus type [F(2,56) = 15.75, *P* < 0.001, η^2^_p_ = 0.36]. Planned comparisons revealed faster responses to the cartoon images (Mean response time = 767 ms) than to the rotoscoped (989 ms; *P* < 0.001) and photo images (956 ms; *P* = 0.001), mirroring our accuracy results. There was no difference between the rotoscoped and photo images (*P* = 1.0), again indicating contrast did not have a singular effect.

These behavioral data clearly show that the cartoon images were overall easier to respond to, even when all the images were presented at 500 ms.

#### ERP data

For all analyses, pairwise contrasts were Bonferroni adjusted for multiple comparisons, and main effects and interactions were corrected using the Huynh–Feldt correction for the violation of sphericity where necessary. For brevity, only significant effects are reported; however, all values for each condition for both P1 and N170 can be found in Tables 2, 3 and 4. Results of the statistical tests performed on the N170 at sensors P9 and P10 can be found in Additional file 1.

**Table 2:** Event-related potential full data (P1) (Oz)

		Magnitude (μv)		Latency (ms)	
Stimulus set	Expression	Mean	Std. Error	Mean	Std. Error
Photo	Disgust	9.314	0.777	124.132	2.560
	Happy	9.409	0.696	125.268	2.418
	Shocked	9.077	0.754	124.269	2.651
	Neutral	9.444	0.668	122.523	2.430
Rotoscoped	Disgust	7.540	0.694	124.571	3.855
	Happy	8.157	0.707	121.875	3.582
	Shocked	8.592	0.701	125.871	3.835
	Neutral	8.411	0.699	124.750	3.735
Cartoon	Disgust	7.136	0.812	125.062	4.431
	Happy	8.071	0.731	128.452	4.416
	Shocked	7.276	0.769	130.221	4.791
	Neutral	8.636	0.796	129.073	4.076

**Table 3:** Event-related potential full data (P1) (P9/P10)

			Magnitude (μv)		Latency (ms)	
Stimulus set	Expression	Sensor	Mean	Std. Error	Mean	Std. Error
Photo	Disgust	P9	3.132	1.844	129.534	19.790
		P10	5.061	2.503	136.998	13.329
	Happy	P9	2.987	1.719	133.371	15.118
		P10	5.171	2.424	137.068	11.803
	Shocked	P9	2.506	1.834	131.208	16.709
		P10	5.085	2.584	133.092	17.066
	Neutral	P9	2.787	1.613	135.603	16.345
		P10	5.112	2.282	135.324	10.892
Rotoscoped	Disgust	P9	3.043	1.521	122.001	15.863
		P10	5.179	2.713	121.652	12.645
	Happy	P9	3.234	1.581	122.140	17.105
		P10	5.056	2.519	120.885	11.997
	Shocked	P9	3.029	1.792	120.675	16.635
		P10	4.990	2.523	122.140	11.987
	Neutral	P9	2.881	1.846	122.419	15.471
		P10	5.179	3.093	121.024	10.717
Cartoon	Disgust	P9	2.623	1.637	117.746	16.563
		P10	4.035	2.776	119.629	15.549
	Happy	P9	2.490	1.654	115.235	16.123
		P10	4.250	2.540	118.234	14.049
	Shocked	P9	2.280	1.500	116.351	18.006
		P10	4.203	3.053	117.327	14.987
	Neutral	P9	2.851	1.659	119.280	12.466
		P10	4.535	2.707	118.164	12.347

**Table 4:** Event-related potential full data (N170)

			Magnitude (μv)		Latency (ms)	
Stimulus set	Expression	Sensor	Mean	Std. Error	Mean	Std. Error
Photo	Disgust	P9	–1.024	0.483	182.509	3.890
		P10	–0.357	0.583	181.858	2.132
	Happy	P9	–0.756	0.451	184.173	3.270
		P10	0.037	0.615	179.470	2.170
	Shocked	P9	–1.273	0.467	187.066	3.017
		P10	0.244	0.606	182.943	2.686
	Neutral	P9	–0.808	0.508	183.883	3.141
		P10	0.007	0.586	182.075	3.058
Rotoscoped	Disgust	P9	–1.195	0.417	172.815	2.705
		P10	–1.504	0.580	166.739	2.621
	Happy	P9	–0.877	0.408	171.007	2.511
		P10	–0.757	0.578	164.786	2.764
	Shocked	P9	–0.981	0.394	170.211	2.885
		P10	–0.708	0.596	167.101	2.357
	Neutral	P9	–1.041	0.448	168.475	2.864
		P10	–0.738	0.552	167.752	2.565
Cartoon	Disgust	P9	–2.192	0.553	174.624	3.514
		P10	–2.354	0.757	168.837	3.130
	Happy	P9	–1.186	0.406	166.956	3.091
		P10	–0.947	0.594	165.003	3.257
	Shocked	P9	–1.726	0.387	173.177	3.096
		P10	–1.666	0.680	165.292	2.699
	Neutral	P9	–0.757	0.450	169.560	3.264
		P10	–0.751	0.471	168.982	2.927

#### P1

**Fig. 5:**
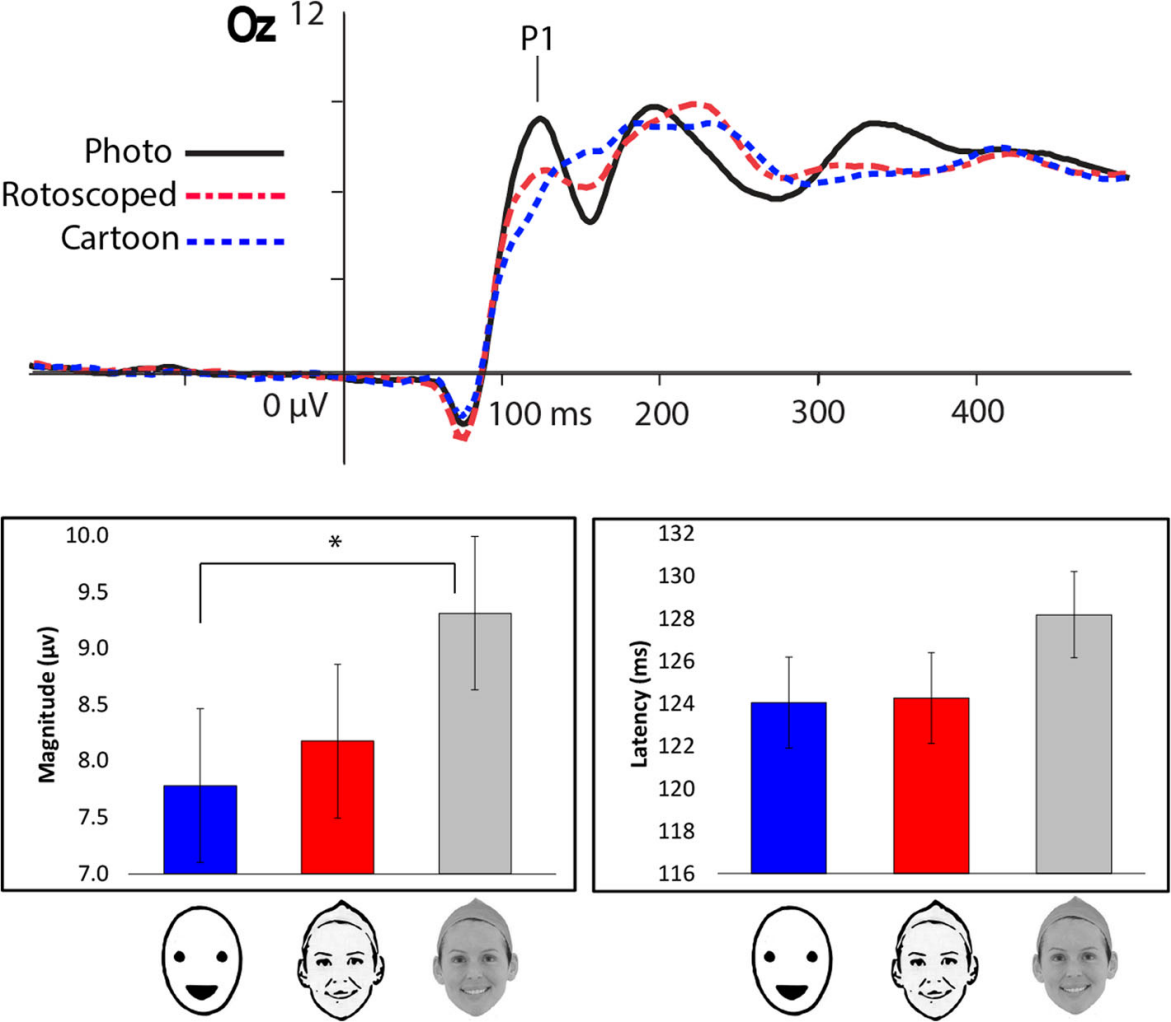
P1 data at sensor Oz for Experiment 2. The waveforms at the top show the three stimulus sets averaged across all participants. The charts below represent the latencies and amplitude of those individual peaks averaged together. * Significant difference at *P* < 0.05

A 3 × 4 (stimulus type × expression) repeated measures ANOVA was performed on P1 amplitude and latency at electrode Oz (Fig. 5). Analysis of P1 amplitude showed a main effect of stimulus type [F(2,50) = 6.13, *P* = 0.004, η^2^_p_ = 0.20]. Follow-up comparisons revealed that amplitudes elicited by cartoon images, which were lowest in contrast and complexity, were significantly lower than those elicited by photographic images, which were highest in both (*P* = 0.021). Comparison of amplitudes elicited by photo and rotoscoped images, as a measure of contrast, revealed only a trend level difference (*P* = 0.07). Comparison of cartoon and rotoscoped images, as a measure of complexity, revealed no difference (*P* = 0.829). These results suggest that, whereas combined reductions of contrast and complexity in cartoon images result in a smaller P1, neither is a singular driving factor in cortical facilitation by iconic images.

Analysis of P1 latency revealed no significant results (*P* > 0.24).

#### Effects of expression

Although we included emotional expression as a factor in our analysis, in accordance with Experiment 1, analysis of expression simply served to illustrate that, for amplitude, the reported effects were slightly stronger for disgusted faces, with no significant effects for latency (*P* > 0.05; Additional file 1).

#### Peak-to-peak analysis

To help understand whether the pattern of P1 results reported above was carried forward into the face-sensitive N170, we performed a follow-up analysis in which we compared the amplitude and latency of the P1 peak with the N170 peak to see if these effects observed in the N170 originated at the P1. A 3 × 4 × 2 (stimulus type × expression × electrode site) repeated measures ANOVA was performed on the peak-to-peak difference between the N170 and the P1 components (for N170 results; Additional file 1).

#### Amplitude

The effect of stimulus type was not significant (*P* = 0.112), although there were some effects of emotional expression (Additional file 1). This suggests that the pattern of results reflects a P1 effect that persists and influences the amplitude patterns observed for the N170. As such, these effects likely do not reflect processes specific to face recognition, but simply the low level featural differences between the stimuli.

#### Latency

None of the main effects or interactions were significant (all *P* > 0.1), further confirming that effects of stimulus-type on the N170 were not face-specific but had an onset at the earlier P1.

## Discussion

The present study examined the hypothesis that iconic faces are processed differently from realistic depictions and communicate emotion more effectively. In Experiment 1, we found that greater schematization in our stimuli, accomplished through increasing contrast and decreasing featural complexity, increased the likelihood a participant would be able to discriminate which emotional expression was present. In addition, both factors increased accuracy in a way that represented separable effects. Notably, at the fastest presentation times, discrimination of expressions in the simplest images had an accuracy rate nearly 60 % higher than in realistic faces, and accuracy was substantially higher than is typically reported at such presentation times (Whalen et al., 1998; Pessoa, Japee, & Ungerleider, 2005).

Experiment 2 revealed that cartoon stimuli evoked distinct patterns of cortical processing from photographic stimuli. Thus, stimuli characterized by both higher contrast and lower levels of featural complexity were associated with lower P1 amplitudes; however, the lack of significant differences between photo and rotoscoped images as a measure of contrast, and cartoon and rotoscoped images as a measure of complexity, suggest neither feature singly accounted for the effect. Together, these two experiments indicate that the greater contrast and simplicity of cartoon images facilitate rapid discrimination of facial emotion associated with reduced need for cortical processing of the images.

The P1 is known to be sensitive to low-level visual features of faces and objects, and is thought to represent an early step in a feed-forward volley of visual stimulus processing, in which information is extracted and passed on to higher-level regions of the ventral visual stream (Woodman, 2010; Rossion & Caharel, 2011; Kappenman & Luck, 2012; Desjardins & Segalowitz, 2013). Our data are consistent with previous findings on pattern-evoked ERPs, where decreased complexity was associated with decreased P1 magnitude (Oken et al., 1987). Recent research has also found that the P1 is magnified for faces above objects even when these stimuli are presented too quickly to be reportable (Mitsudo, Kamio, Goto, Nakashima, & Tobimatsu, 2011). This finding provides further convergent evidence that the advantage in discriminating information from more iconic images, based on low-level features, is heightened when images are presented at the threshold of detection and would first be observed at the latency of the P1. Such an advantage may then contribute to later configural processes that may modulate the face-sensitive N170 (Rossion & Caharel, 2011). This may help describe how previous work has found more negative N170 amplitudes for emoticons than photographs (Churches et al., 2014); while we replicate this effect with our cartoon stimuli, our findings indicate that this N170 modulation is not N170 specific, but has an onset much earlier at the P1.

Together with the above previous studies, the results of our experiments tell a story, namely that increased contrast and complexity enhance the P1, which is a measure of relatively early perceptual processing of stimulus features. This enhancement can be seen as propagating forward into later stages of face-specific processing, and are observed in the N170 peak as an ongoing reflection of featural differences. This is then reflected in our behavioral results as decreased accuracy and (in Experiment 2) longer reaction times. Our finding that differences between conditions observed in the N170 originated with the P1 is informed by a longstanding discussion about the role of the P1 in face processing.

P1 effects that precede and correspond to later N170 effects have been frequently observed – for example, contrast reversal and inversion have been found to affect P1 as well as N170 latency and magnitude (Taylor, 2002; Itier & Taylor, 2004). The caveat to such findings is that inverting the contrast or spatial orientation of a face image, as with any other image, is directly influencing the stimulus’ low level features in a manner similar to this study. The simplest explanation is that the changes in P1 amplitude found here and in previous studies represents a difference in availability of information in a given stimulus, which can facilitate subsequent processing. Our findings suggest that exaggeration of low-level features provides the basis for more efficient extraction of facial information in general, including information about facial identity (e.g., caricatures). It is also possible that the effects of schematization in facilitating facial emotion identification may not be specific to face processing but may generalize to ease of information extracted from any type of stimulus with higher contrast and simpler features.

It is important to note, however, that in creating images with low complexity, we also reduced stimulus variability and such reduced variability may also have influenced our results. Despite efforts to add variability to our cartoon stimuli, it can be difficult to mirror the natural variability in photographic faces in the medium of cartoons. Future research can investigate questions of stimulus variability as a contributing factor to the effects of iconic stimuli. From the results found in this study, however, the question arises of whether “schematization” can also enhance the efficiency of object discrimination, or whether it bestows a special advantage to configural processes that are particularly important for discrimination of facial identity and emotion.

In practice, contrast and featural complexity are frequently manipulated together such as in public environments when information must be detected quickly across contexts (e.g., Babbitt Kline, Ghali, Kline, & Brown, 1990). However, graphic emoticons have decreased in contrast since their inception (e.g., color emoticons have lower contrast than their ASCII equivalents) but remain featurally simplistic, underscoring the importance of this factor. Aside from simplicity, emoticon features vary widely, and are still widely used for modulating textual emotional content (Luor, Wu, Lu, & Tao, 2010; Rojas, Kirschenmann, & Wolpers, 2012). The communicative role of other features, such as color, in iconic representations is another interesting avenue for research. Yet, other questions to be pursued include, but are not limited to, whether other factors (e.g., emotional intensity) were manipulated secondarily along with contrast and featural complexity, and if so, what, if any role do they play in the present results. Similarly, in our study, we tested low-level features that varied qualitatively, and so there could be effects of contrast or featural complexity that could only be found by scaling both low-level features parametrically. Further research will be required to investigate this possibility.

## Conclusions

Iconic faces can be viewed either as analogous to realistic images or as a distinct class of stimulus. Our findings support the view that iconic representations serve a distinct role – to impart specific information quickly and efficiently – and highlight the advantages of simplifying image features and increasing contrast to communicate emotion. In addition, our data suggest that the effects of iconization may not be specific to faces, but rather to any stimulus that has these low-level featural changes. It is thus important to consider that such features are not just potential low-level confounds but contribute to specific communicative functions. However, it is unknown if the discrimination of more subtle real-world types of emotional expression would also benefit from iconic representation (e.g., the ‘Duchenne’ smile, where genuine happiness is expressed with the wrinkling of the corners of the eyes) (Ekman, Davidson, & Friesen, 1990). It may be that iconic images have a communicative advantage only for simple visual information, a hypothesis that invites future research.

The effective communicative role of iconic images may underlie the ubiquity and popularity of iconic imagery and cartoons in popular culture. Better understanding of the factors that enhance their communicative role may help improve their use in various real-world applications such as emoticons, signs, and concept cartoons. We suggest that the communicative role of iconic imagery is an important area for further research, and its power would be better exploited than ignored.

## Additional file

Additional file 1: Effects of emotional expression and excluded results. (PDF 206 kb)
