# Supplementary material for: Iconic faces are not real faces: enhanced emotion detection and altered neural processing as faces become more iconic
Source: Cogn Res Princ Implic. 2016 Dec 12;1:19. doi: 10.1186/s41235-016-0021-8 (PMC5256461; doi:10.1186/s41235-016-0021-8)
Supplement: Supplementary file 1 — Effects of emotional expression and excluded results. (PDF 206 kb) [file 41235_2016_21_MOESM1_ESM.pdf]

Appendix

Effects of emotional expression and excluded results

Experiment 1

There was a main effect of expression type [$F(3,138)= 12.42$, $p<.001$, $\eta^2_p = .21$], suggesting that some emotions may be easier to process than others. However, follow-up comparisons revealed that this was driven purely by disgust, which was significantly different from all the other emotions (all ps<.001, with the exception of the comparison with neutral expression, $p=.032$). No other expressions differed from each other (all ps>.5). This likely simply indicates that the pattern of results was magnified for disgust compared to the other emotions.

There was also an interaction between level of schematization and expression [$F(12,552)= 6.27$, $p<.001$, $\eta^2_p = .12$]. Planned contrasts revealed this interaction was driven by faster identification of disgust faces at shorter presentation times.

Finally there was a three-way interaction between stimulus type, expression, and presentation time [$F(36,1656)= 11.55$, $p<.001$, $\eta^2_p = .20$]. This interaction was driven once again by an exaggerated profile for disgust compared to the other expressions (the difference in accuracy between the disgust and other stimulus categories was wider at the faster presentation times).

Experiment 2

Behavioral

For accuracy, there was a main effect of expression type [$F(2.4, 66.64)= 9.08$, $p<.001$, $\eta^2_p = .25$]. While the disgust expression was different from happy ($p=.04$), this effect was primarily

driven by the shocked expression differing from both happy ($p<.001$) and neutral ($p=.001$). No other comparisons were significant ($ps>.250$). The shocked faces may have seemed more ambiguous, as at 92.9%, they were over 3% lower than happy (97.4%) and neutral (96.5%) faces.

There was also an interaction between stimulus type and expression type [$F(4.39,122.90)= 6.74$, $p<.001$, $\eta^2_p = .194$]. Follow up contrasts revealed that this interaction was driven by the relative flatness of cartoon images (all ps =1.0, with a range of 98.0 – 98.5%) compared to the other stimulus categories. For the rotoscoped images, the shocked expressions were significantly lower (90.0%) than the happy (96.8%) and neutral images (95.2%) (ps= .001 and .01 respectively). Similarly, for photos, the shocked expressions (90.1%) were significantly lower than the happy (97.2%) and neutral (96.2%) images ($ps<.001$). However here the disgust faces (92.4 %) were also low enough compared to the happy faces to be significant ($p=.048$).

Altogether this pattern of accuracy to emotional expressions suggested that the shocked and disgusted faces were easier to mistake than the happy or neutral, or that, more likely, with more realistic faces there is simply a bias to responding happy or neutral versus shocked or disgusted.

For reaction times, there was also a main effect of expression type [$F(3, 84)= 4.41$, $p=.006$, $\eta^2_p = .14$]. Planned comparisons revealed that this was driven by happy images being responded to faster than disgust ($p=.024$) or shocked ($p=.012$) images.

There was no interaction between stimulus type and expression type ($p=.475$).

P1 - Oz

Expressions

For P1 amplitude at electrode Oz, there was also a main effect of expression [$F(3,75)= 4.83$, $p<.003$, $\eta^2_p = .16$]. Follow up comparisons revealed that this was driven by the unique pattern of disgust faces, which were significantly different from shocked faces ($p=.02$) and neutral faces ($p=.01$). No other comparisons were significant ($ps>.37$). There was also a significant interaction of expression type and stimulus type [$F(4.66,116.48)= 2.58$, $p=.033$, $\eta^2_p = .09$].

There were no significant expression effects on the P1 for latency at Oz, or for amplitude or latency at P9/P10 ($ps>.05$).

P1 – P9/P10

Analysis of P1 amplitude revealed a main effect of stimulus type [$F(1.56,42.16)= 7.83$, $p=.003$, $\eta^2_p = .23$]. Follow up comparisons revealed that cartoon images evoked a lower amplitude P1 than photos ($p=.049$) and rotoscoped ($p<.001$) images, but that photo and rotoscoped images did not significantly differ.

There was also a main effect for the sensor site at which P1 was recorded [$F(1, 27)= 21.60$, $p<.001$, $\eta^2_p = .44$], with P10 showing a larger P1 than P9.

Analysis of P1 latency revealed a main effect of stimulus type [$F(1.44, 38.87) = 48.03$, $p<.001$, $\eta^2_p = .64$]. Pairwise comparisons revealed that latencies for each of the three stimulus types different from each other (all $ps <.01$). Cartoon images evoked the shortest latencies, followed by rotoscoped images, and photos evoked the longest latencies. No other main effects or interactions were significant (all $ps>.1$)

N170 – P9/P10

Amplitude

Analysis of N170 amplitude revealed a main effect of stimulus type [$F(1.63,42.29)= 8.88$, $p=.001$, $\eta^2_p = .25$] (see Figure 5). Contrasts revealed that the N170 for the cartoon stimulus set was larger than both the rotoscoped and photo sets ($p=.017$ and $p=.004$, respectively), but that these latter two sets did not differ from each other ($p=.167$). Because the rotoscoped images and the cartoon images share contrast, this finding suggests that featural simplicity of the cartoon stimulus set rather than differences in contrast underlie differences in N170 amplitude, which matches the differences found in the P1 analyses.

There was also an interaction between electrode and stimulus set [$F(2, 52)= 14.05$, $p=.03$, $\eta^2_p = .13$]; Follow-up ANOVAs at each electrode site (i.e., one for P9 and one for P10) revealed that this is because, while the main effect of stimulus set was in the expected direction, it was not significant at P9 when that electrode was considered alone ($p=.11$); in contrast, the effect was robust at P10 [$F(2, 56)= 11.19$, $p<.001$, $\eta^2_p = .29$].

Amplitude (expressions)

There was also a main effect of expression type [$F(3,78)=12.58$, $p<.001$, $\eta^2_p = .33$]. Pairwise comparisons revealed that the disgust images evoked a larger amplitude N170, which differed from that for all other expressions ($ps>.05$), which in turn did not differ from each other (all $ps > .2$).

There was an interaction between stimulus set and expression type [$F(6, 156)= 5.07$, $p=.009$, $\eta^2_p =.10$]. Planned contrasts revealed that the main effect of stimulus set was not significant for the neutral expression stimuli ($p=.143$) but was significant for all other

expressions (all Fs > 4.0, all ps< .05), suggesting that the advantage of featural complexity may have a greater advantage in emotional (i.e., non-neutral) stimuli.

Latency

Analysis of N170 latency revealed a main effect of stimulus type [$F(2, 52) = 59.46$, $p<.001$, $\eta^2_p = .70$]. Contrasts revealed differences in latency between the N170 for photos and both rotoscoped and cartoon stimuli (ps <.001). The rotoscoped and cartoon stimuli evoked an N170 that was, on average, 13.9 ms faster than the photo stimuli.

Latency (expressions)

We observed a main effect of expression type [$F(3, 78)= 2.981$, $p=.036$, $\eta^2_p = .10$]. Pairwise contrasts revealed that, although happy expressions were processed slightly faster than other expressions (on average 1.56 - 2.66ms), there were no significant latency differences between pairs of expression type (ps>.05).

Peak to Peak

Expressions

A main effect of expression was found [$F(2.25, 62.03)= 19.49$, $p<.001$, $\eta^2_p =.44$]. Pairwise comparisons revealed that this was driven by the disgust faces showing a larger difference than all other stimulus types (ps<.001). This further supports our disgust stimuli as being processed uniquely, but as this did not vary with stimulus type, this information is tangential to our hypothesis.

There was also peak to peak interaction of stimulus type and emotional expression, [$F(6, 150)= 3.63$, $p=.002$, $\eta^2_p =.13$]. However, in follow up analyses this interaction seemed to be driven solely by a significantly greater N170/P1 difference for happy faces only between rotoscoped and photo stimulus sets ($p=.044$).
